# Complete plastome sequence of *Eustoma grandiflorum* (Gentianaceae), a popular cut flower

**DOI:** 10.1080/23802359.2019.1667893

**Published:** 2019-09-23

**Authors:** Jingyan Yan, Qian Cao, Zesi Wu, Shaofeng Chen, Jiuli Wang, Dangwei Zhou, Jiuxiang Xie

**Affiliations:** a State Key Laboratory of Plateau Ecology and Agriculture, College of Agriculture and Animal Husbandry, Qinghai University, Xining, Qinghai, China;; b Key Laboratory of Adaptation and Evolution of Plateau Biota, Northwest Institute of Plateau Biology, Chinese Academy of Sciences, Xining, China;; c Key Laboratory of Biotechnology and Analysis and Test in Qinghai-Tibet Plateau, College of Ecological Environment and Resources, Qinghai Nationalities University, Xining, China

**Keywords:** *Eustoma grandiflorum*, plastome, lisianthus, Gentianaceae

## Abstract

*Eustoma grandiflorum* (Raf.) Shinners is a popular cut flower due to its beautiful morphological characteristics and extended vase life. Here, the complete plastome sequence of *E. grandiflorum* was reported based on the Illumina HiSeq Platform. The plastome sequence is 154,230 bp in length with a typical quadripartite structure, containing a pair of inverted repeated (IR) regions (25,755 bp) that are separated by a large single copy (LSC) region of 84,297 bp, and a small single copy (SSC) region of 18,423 bp. A total of 130 functional genes were annotated, including 84 protein-coding genes, 38 tRNA genes, and 8 rRNA genes. The complete plastome sequence of *E. grandiflorum* will provide a valuable resource for its garden utilization and the phylogenetic studies of Gentianaceae.

*Eustoma grandiflorum* (Raf.) Shinners, known as lisianthus, is a perennial herbaceous of Gentianaceae. *E. grandiflorum* is more and more popular because of its beautiful morphological characteristics and extended vase life (Davies et al. 1993). Plastids support the survival of a large number of creatures on Earth by providing food, oxygen, and fuel. The plastome has also been smartly engineered to confer valuable agronomic traits (Jin and Daniell 2015). However, no studies on the plastome of *E. grandiflorum* have been published. In this study, the complete plastome of *E. grandiflorum* (Genbank accession number: MK991810) was sequenced on the Illumina HiSeq Platform, which will provide genetic and genomic information to promote its garden utilization and systematics research of Gentianaceae.

In this study, *E. grandiflorum* were sampled from Xiaohe village, Daojiao town, Dongguan city, Guangdong Province China (23.03°N, 113.66°E) and the fresh, young leaves were dried immediately by silica gels. Total genomic DNA of *E. grandiflorum* was extracted from the dried leaves (about 0.2 g) with a modified CTAB method (Doyle and Doyle 1987). The voucher specimen was kept in Herbarium of the Northwest Institute of Plateau Biology, Northwest Institute of Plateau Biology, Chinese Academy of Sciences (HNWP, Wang2018001). Genome sequencing was performed using the Illumina HiSeq Platform (Illumina, San Diego, CA, USA) at Genepioneer Biotechnologies Inc., Nanjing, China. Approximately 6.59 GB of clean data were yielded. The trimmed reads were mainly assembled by SPAdes (Bankevich et al. 2012). The assembled genome was annotated using CpGAVAS (Liu et al. 2012).

The complete plastome of *E. grandiflorum* is 154,230 bp in length with a typical quadripartite structure, containing a pair of inverted repeated (IR) regions (25,755 bp) that are separated by a large single copy (LSC) region of 84,297 bp, and a small single copy (SSC) region of 18,423 bp. The GC content of the whole cp genome was 37.92%. A total of 130 functional genes were annotated, including 84 protein-coding genes, 38 tRNA genes, and 8 rRNA genes. The protein-coding genes, tRNA genes, and rRNA genes account for 64.62, 29.23, and 6.15% of all annotated genes, respectively.

Phylogenetic relationships of *E. grandiflorum,* with 19 other species of Gentianaceae and *Cynanchum auriculatum* (Asclepiadaceae) were resolved by means of Neighbor-joining (Figure 1). The alignment was conducted using MAFFT (Katoh and Standley 2013). The Neighbor-joining tree was built using MEGA7 (Kumar et al. 2016) with bootstrap set to 1000. The phylogenetic tree showed that *E. grandiflorum* is far from the other species of Gentianaceae that with plastome sequence known. The complete plastome sequence of *E. grandiflorum* will provide a valuable resource for its garden utilization and the phylogenetic studies of Gentianaceae.

**Figure 1.**
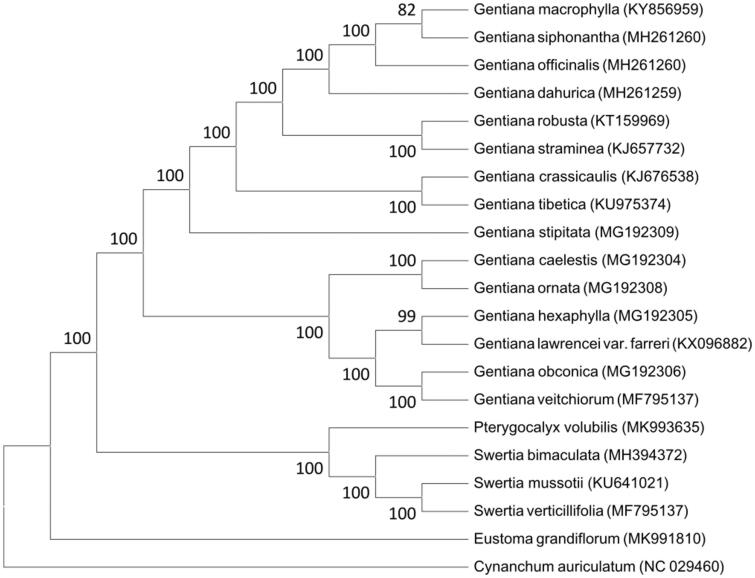
The neighbor-joining tree based on 21 plastome sequences.
